# Nanoscale Assembly of Copper Bearing-Sleeve via Cold-Welding: A Molecular Dynamics Study

**DOI:** 10.3390/nano8100785

**Published:** 2018-10-04

**Authors:** Hongjian Zhou, Jiejie Li, Yuehui Xian, Guoming Hu, Xiaoyong Li, Re Xia

**Affiliations:** 1 Key Laboratory of Hydraulic Machinery Transients (Wuhan University), Ministry of Education, Wuhan 430072, China; jackzhou@whu.edu.cn (H.Z.); jjlee@whu.edu.cn (J.L.); xianyuehui@whu.edu.cn (Y.X.); hu_guoming_cn@163.com (G.H.); 2 Hubei Key Laboratory of Waterjet Theory and New Technology (Wuhan Unijversity), Wuhan 430072, China

**Keywords:** bearing, cold-welding, nano-assembly, mechanical structure, molecular dynamics

## Abstract

A bearing is an important component in contemporary machinery and equipment, whose main function is to support the mechanical rotator, reduce the friction coefficient during its movement, and guarantee the turning accuracy. However, assembly of a nanoscale bearing and sleeve is a challenging process for micro-nano mechanical manufacturers. Hence, we show the cold-welding mechanism of a copper nanobearing-nanosleeve via molecular dynamic simulations. We demonstrate that it is feasible to assemble a bearing and sleeve at the nanoscale to form a stable mechanism. The effect of temperature in the range of 150 to 750 K is investigated. As the temperature rises, the mechanical strength and the weld stress of the welded structures markedly decrease, accompanied by the observation of increasing disorder magnitude. This comparison study is believed to facilitate future mechanical processing and structural nano-assembly of metallic elements for better mechanical performance.

## 1. Introduction

Metallic nano-components (for example, nanowires) have been studied broadly due to their great potential use in many fields, such as electronics, optical engineering mechanics, optical science, and material science. However, few studies have focused on the combination of the two mechanical components (bearing and sleeve) at the nanoscale via cold-welding. A bearing, as a machine element, reduces friction between moving parts and constrains relative motion only to the desired motion. The design of the bearing provides for free linear movement of the moving part or for free rotation around a fixed axis; or it may prevent motion by controlling the vectors of normal forces that bear on the moving parts. Molecular Dynamic (MD) simulation is an essential scientific tool for studying mechanics and nanomaterial interaction. Numerous nanosystems have been researched using MD simulation, such as nanoforming [1], nanodrilling [2], additive manufacturing [3,4], and mechanical behavior [5,6].

The nanoscale cold-welding technique is convenient to assemble and join simple constructions under the relatively easily available conditions of low applied pressure and low temperature. In 2010, Lu et al. [7] elucidated the underlying mechanism of nanoscale cold-welding by head-to-head joining of gold nanowires (NWs). From then on, cold-welding has been considered as the burgeoning bottom-up nanofabrication and nanoassembly techniques with great promise. In recent years, most studies focused on the cold-welding mechanism considering various material phases [8,9,10,11,12,13,14], welding conditions [15,16] and joining procedures [17,18].

This work investigates the nanoscale assembly of a copper nanobearing and nanosleeve via the cold-welding method using MD simulations. The results are discussed from several aspects such as common neighbor analysis (CNA), longitudinal stress, radial distribution function (RDF), and atom displacement vectors. To verify the quality of the welding joints and evaluate their mechanical strength, assembled structures are stretched continuously with a constant rate until rupture. Owing to its difficulty or near impossibility of experimentally capturing the evolution of atomistic structure under continuous loading, our study is aimed at providing the foundation for future experiments and applications [12].

## 2. Theoretical Methods

### 2.1. Modeling of Copper Bearing-Sleeve

**Figure 1:**
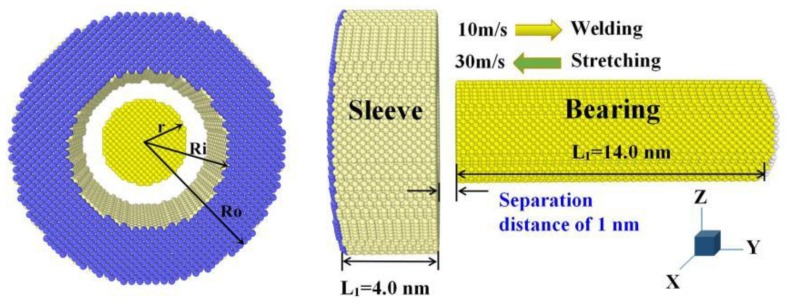
General scheme for the MD (molecular dynamic) welding mode between nanobearing and nanosleeve.

There are two parts of the initial model structure (Figure 1). The first part is a nanobearing which is 6 nm in radius for the outer ring (R_o_) and 2.5 nm radius for the inner ring (R_i_), and the second part is a nanosleeve which is 2.5 nm in radius (r) and a length of 14 nm. To avoid early interaction at the stage of thermal equilibrium, we set a separation distance of 1 nm between the two components before cold-welding begins. The whole sample contains approximately 5.6 × 10^4^ atoms. In the next step, to minimize stress in the atomic model, the temperature was held at 300 K using a Nose-Hoover thermostat [19]. Lastly, the simulations entered into the welding stage after full relaxation.

### 2.2. Computational Methods and Parameters

Large-scale MD simulations of cold-welding and stretching processes are performed on nanobearing-nanosleeve using the Atomic/Molecular Massively Parallel Simulator (LAMMPS) package [20] with a time step of 0.005 ps. Based on our previous work [12,13], the Finnis-Sinclair embedded-atom method (EAM/FS) for Cu by Mendelev [21] was adopted to describe the interatomic interactions, structure, energy, and dynamics of large scale defects with reasonable accuracy. In the EAM/FS, the total energy is given by:

(1) $$E_{i}=F_{\alpha }\left(\sum_{j\neq i}\rho_{\alpha \beta }\left(r_{ij}\right)\right)+\frac{1}{2}\sum_{j\neq i}\Phi_{\alpha \beta }\left(r_{ij}\right)\;$$

where the subscripts *i* and *j* label distinct atoms, *r_i,j_* is the separation between atoms *i* and *j*, *F* is the embedding energy, and α and β are the element types of atoms *i* and *j*. ρ is the pair potential contribution to the cohesive energy and Φ represents the energy to embed an atom in a background charge density. This has the same form as the EAM formula, apart from that the atomic electron density is a function specific to the atomic types of both atoms *i* and *j*.

To further analyze the results, stress–time curves are obtained by exact accumulation and calculating the longitudinal stress on the system. The common neighbor analysis (CNA) [22] are utilized to help analysis of the defect structures before and after deformation during the simulation. Images in this paper were created using the Open Visualization Tool (OVITO) package [23].

### 2.3. MD Simulation Details

This MD simulation can be divided into three stages: the welding stage, the holding stage, and the stretching stage. The first process lasts 200 ps, and we apply the constant rate of 0.1 Å per 1 ps (that is 10 m/s) along the y-axis to achieve the desired results. Next is the holding stage, where the welding velocity is set to 0. Finally the welded structure goes through the stretching stage until it fractures, to examine its mechanical properties. In this study, no periodic boundary conditions are applied in all dimensions to simulate nanobearing and nanosleeve of finite length. MD simulations at different temperatures are performed to investigate how this affects the atomic diffusion. The nanobearing and nanosleeve with the same length but with a different inside radius of nanobearing are researched as well as for contrast tests. The comparison is made based on the scenarios below which are summarized in Table 1: (i) nano-assembling between nanobearing and nanosleeve with the same length but of different inside radius; (ii) the simulation is performed from 150 to 750 K with temperature increments of 150 K; and (iii) the moving rate and stretching is changed with various values.

## 3. Results and Discussion

**Figure 2:**
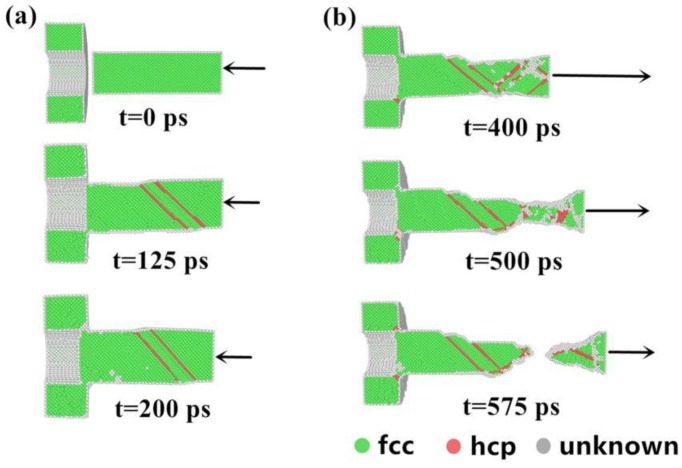
Structural evolution of Cu-Cu through common neighbor analysis (CNA) assembly for various times: (**a**) 0, 125 and 200 ps; (**b**) 400, 500 and 575 ps.

As the cold-welding proceeded as shown in Figure 2a; the structural evolution sequence was displayed for the assembling process of the Cu nanobearing and nanosleeve at different points in time (0, 100, and 200 ps) at a constant rate (10 m/s). Atoms were colored green, red, and gray to denote their belonging to face-centered-cubic (FCC), hexagonal-close-packed (HCP), and disordered environments (or surface lattice), respectively. At the beginning of the simulation, nanobearing on the right side moved to the left nanosleeve until the two components reached the point of contact (see Figure 2a). The two components firstly came into contact with an incomplete jointing area after around 125 ps and then they connected and integrated by a gradually growing bonding-area until the withdrawal of the loading. Both nano-components kept the same crystal structure during the holding time (200–300 ps) when the velocity was set at zero. It might be easier to overcome the diffusion energy barrier for a Cu atom at ambient temperature due to its low value (less than 1 eV, typically) [7,24]. Obviously, the activity of atoms at the nanostructure metal surface is clearly better than that in the bulk counterpart. The forming of cold-welding occurs easily on account of the aforementioned two factors: Lower diffusion barrier and higher atom activity.

After the holding process (t = 300 ps), the welded structure was stretched at a certain velocity value (30 m/s) until fracture to examine the quality. Figure 2b displays the structural evolution sequence of the assembled structure during the stretching process at various points in time (400, 500, and 575 ps), which was sketched at a plane cut perpendicular to the direction of cold-welding to detect the change processes of each atom. Under the action of a large tensile force, successive deformation occurred around the necking area with further stretching, nevertheless most of the other areas exhibited no significant variation. Next, the necking of nanobearing gradually occurred and was located in the lower strength zone remote from the weld interface. Figure 2b clearly describes the process of how the internal structure evolves from the beginning of the stretching process to the breaking point.

**Figure 3:**
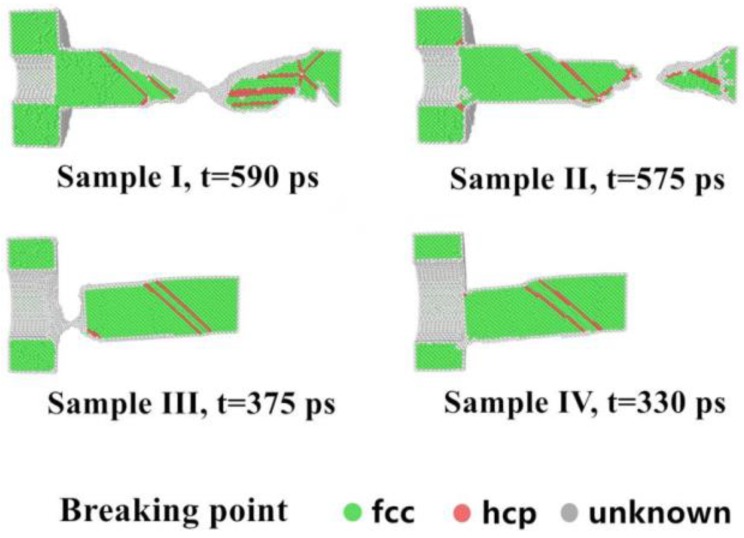
Snapshot of welded structures when rupture occurred for Samples I, II, III and IV (fcc, face-centered-cubic and, hcp, hexagonal-close-packed).

To test the effect of inside radius on assembling performance, Cu nanobearing with different inside radiuses of 2.0, 2.5, 3.0 and 3.5 nm were simulated in the same conditions of 10 m/s (welding velocity) and 300 K. This effect is obvious with the changing inside radius as shown in Figure 3. Figure 3 shows snapshots of the welded structure with Sample I to IV at a time of 590, 575, 375, and 330 ps before the breaking point, respectively. As can be seen in the diagram, the distribution of the breaking points varies with the inside radius, as this effect leads to the different cold-welding surface area. Specifically, the breaking point of Sample I and II occurred at the nano-bearing area, which demonstrated the mechanical performance of welding joints is better than in other areas. Results show that high stress concentration around the fracture areas may be the determining factor for formation of the unstable structure (HCP structure) before the break.

**Figure 4:**
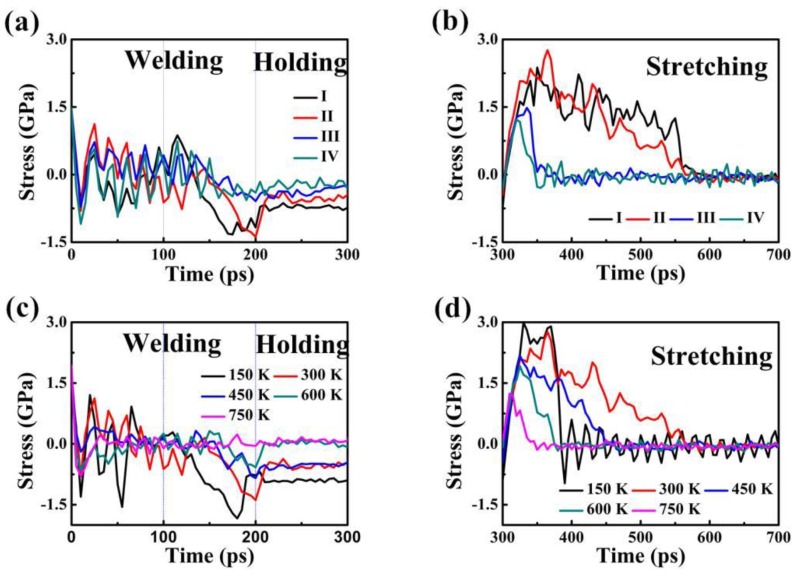
Stress-time curves at longitudinal direction of Sample I, II, III and IV assembled structures at time of (**a**) 0 to 300 ps and (**b**) 300 to 700 ps. Stress-time curves of Cu–Cu nanobearing-nanosleeve structures versus time at different temperatures (150–750 K) during (**c**) 0 to 300 ps (**d**) 300 to 700 ps.

Based on Figure 4a,b, the tendency of stress-time responses is similar in the four control groups. Calculating by collecting all the atom’s longitudinal stresses and taking the average leads to the longitudinal stresses in the nanobearing-nanosleeve pair. Stress-time curves fall into three periods: welding (0–200 ps), holding (200–300 ps), and stretching (rest of time) until the structure fractures. The stress value is not high and therefore few defects form in welding. It is the rapid stress relaxation through the structure self-adjustment after the cold-welding process that results in values below 3.0 GPa. The results show that relatively low stress values lead to a much smaller number of defects forming. As displayed, the linear region of the curves indicates that welded structures firstly experience a short elastic deformation when the stretching process (after 300 ps) begins. The results show that the yielding stress of structures was computed to be 2.37, 2.55, 1.48, and 1.16 GPa for Sample I to IV, respectively. This is due to the effect of the inside radius on the strength of the welded joints. After reaching the curve peak, heavy plastic deformation occurred, and the stress curves do not coincide with each other for the occurrence of various rupture behaviors. The four stress responses have an entirely uniform trend, and the numerical value decreased as time elapsed, and approached zero.

The results of the three stages (welding, holding, and stretching stage) of the assembled structure based on the stress versus time curves are represented in Figure 4c,d, for different temperatures (150, 300, 450, 600, and 750 K). Stress values in the period of time from welding to holding are relatively low, similar to the initial structures, suggesting that the amount of defects formed is small. At a time of 200 to 300 ps, the stress relaxed rapidly via structural self-adjustment, which led to the gradually flattening curves. It is natural to perceive the fluctuations in curves with a zigzag pattern [9], because of the nucleation and activities of dislocation in the period of time (200–300 ps) step-by-step release the accumulated stress. When the stretching process (after 300 ps) starts, the stress-strain curves rise linearly in the first stage and then the rest of the curve exhibits a negative slope. The stress value diminished sharply, which reveals a linear decreasing trend along the time approximately ranging from 320 to 350 ps. The stress value decreases slowly and tends to stabilize at zero, which presents as a marked serration. Clearly, the stress decreases as the temperature increases, suggesting that the pressure required for welding is markedly reduced because of increasing heat. The yield strength of the welded structure presents as a downtrend with increasing temperature, being 1.24 (T = 150 K), 1.92 (T = 300 K), 2.17 (T = 450 K), 2.34 (T = 600 K) and 2.99 GPa (T = 750 K), respectively. 

**Figure 5:**
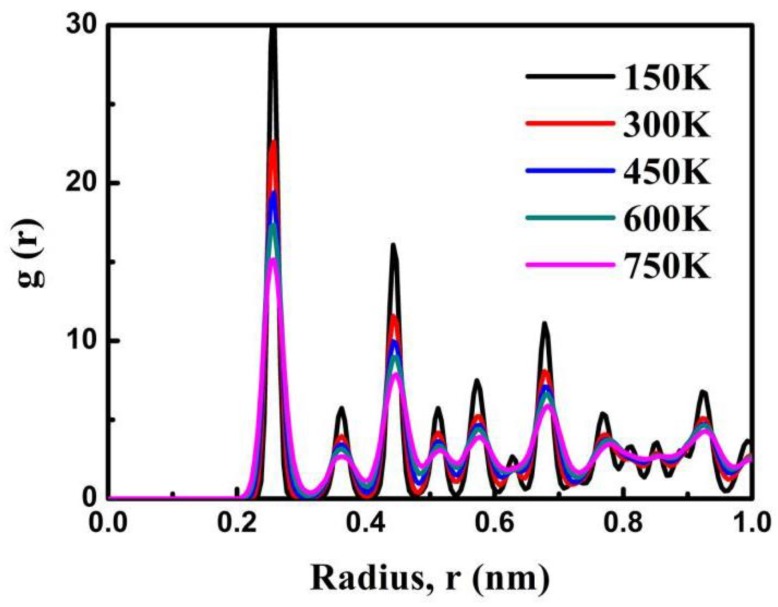
Radial distribution functions after the holding process under the different temperatures conditions (150, 300, 450, 600 and 750 K).

**Figure 6:**
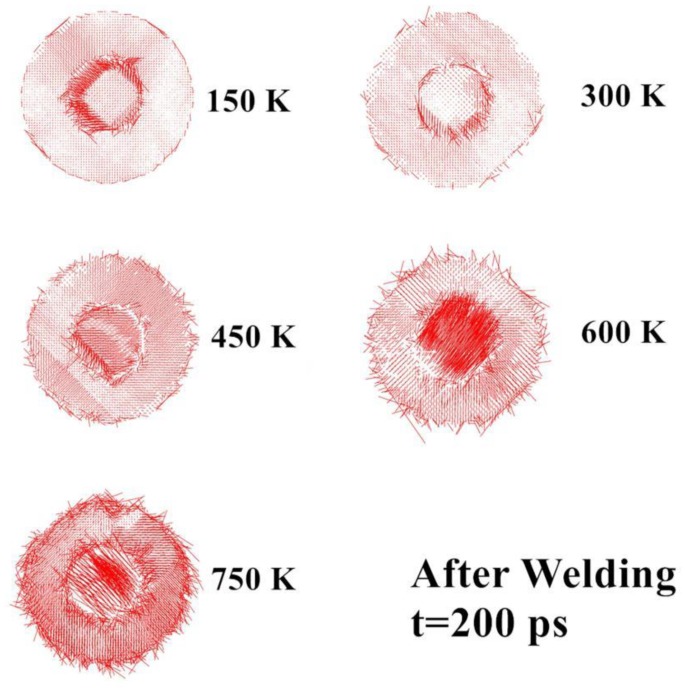
Atoms diffusion for temperatures of 150–750 K after holding (t = 300 ps) are shown by atom displacement vectors.

To further characterize the structure of the Cu nanobearing-nanosleeve structure, the radial distribution function (RDF) has been evaluated. Figure 5 shows the RDF at different temperatures after the holding process (t = 300 ps). With the increasing of temperature, the *g*(r) peak value slowly rises which suggests the emergence of a more well-ordered structure under a relatively lower temperature. When the temperature is decreased from 750 to 150 K with temperature increments of 150 K, the first *g*(r) peak value increased from 15.1 to 17.3, 19.4, 22.6, and 31.5, respectively. Specifically, it is shown that higher temperatures are accompanied to turn disorder and split ascribed to high atomic kinetic energies [17], and hence we become conscious of the emergence of disordered structures in large quantity at temperature of 750 K. Atoms diffusion for temperatures of 150–750 K after holding are shown in Figure 6 by atom displacement vectors. It is clear from Figure 6 that rising temperatures improve atoms diffusion and atoms present are disordered. Nevertheless, the cold-welding process is prevented by excessive temperature during the molten phase [14].

**Figure 7:**
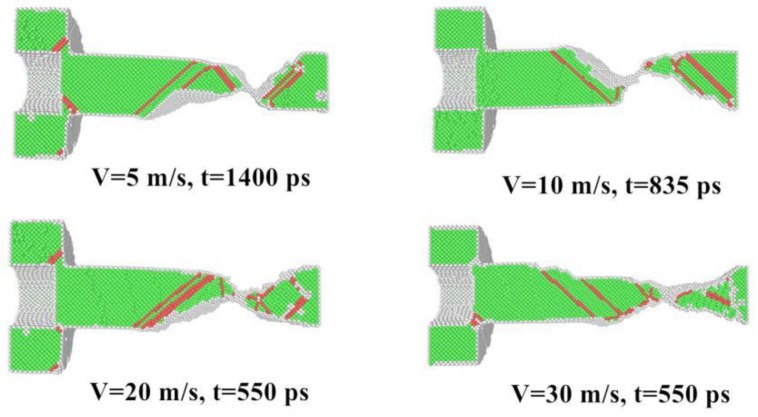
CNA graph for different stretching velocity before the fracture.

On this basis, we further investigate the effect of cold-welding velocity on the assembled structure. As Table 2 indicates, changes in crystal structure is evident. The fraction of FCC atoms is 75.9% and there is HCP atoms of 3.0% at a pace of 10 m/s. When the cold-welding rate reaches 30 m/s, the fraction of FCC and HCP atoms are 72.0% and 6.0%, respectively. This phenomenon indicates that a higher loading rate leads to the emergence of a composition with more disorganized structures. Hence, the structure’s property change is ascribed to the increase in kinetic energy as the cold-welding rate goes up. In the same way, we consider the factor of stretching velocity whether it affects the position of the rupture. It is clearly observed in Figure 7 that the fracture occurs in the base metal rather than in the welded zone. This could be additional evidence for better mechanical performance of the welding joint.

Compared with the interference fit at the macroscopic scale, nanoscale assembly offers advantages. An interference fit is a fastening between two parts which is achieved by friction after the parts are pushed together, rather than by any other means of fastening. There are two basic methods for assembling an oversize shaft into an undersized hole which are force and thermal expansion or contraction [25]. The disadvantage of interference fit is the difficulty of assembly. Alberdi and co-authors researched how process parameters of kerf geometry affect abrasive water jet milling, which provides a way for reference for assembly of bearing and sleeve on a macro-scale [26]. Cumings and Zettl showed that the controlled and reversible telescopic extension of multiwall carbon nanotubes, thus realizing ultralow-friction nanoscale linear bearings and constant-force nanosprings as early as 2000 [27]. Similarly, Zhang proposed a novel nanobearing through the physical adsorption of the confined fluid to the solid wall [28]. In contrast, assembly technology of cold-welding possesses such desirable conditions: a lower stress and temperature. Here, the simulations results show the nanobearing-nanosleeve joining procedure give similar effects that clearly show that temperature affects the assembling and it is possible to bond two components at various temperatures. This joining procedures is other than welding between metal nanowires [8,9,11,13] or polycrystalline metals [10,12], for the welding geometries is side-to-side instead of head-to-head. The diffusion barrier for a single metal atom on a metal surface is as low as less than 1 eV [7,24]. Thus, even low mechanical pressure can help the cold-welding of Cu nano-components to overcome such low barriers at ambient temperature.

## 4. Conclusions

This research studied the process of assembling a Cu-Cu nanobearing-nanosleeve and the temperature effect on the mechanical performance of cold-welding of the Cu-Cu assembled structure. The stress-time responses suggested that both components were successfully bonded through small loadings. The results from the research demonstrates that it is possible for cold-welding of a nanobearing and nanosleeve to form a new mechanical structure at the nanoscale. Then, continuous tensile velocity was applied along the length direction to stretch until breaking. Therefore, we may safely arrive at the following conclusions:

1. The inside radius of the nanobearing affects the mechanical performance because of joint size.
2. Mechanical strength and weld stress of combination observably go down as temperature rise, due to the formation of relatively fewer quantity disordered structures when cold-welding.
3. A higher loading rate lead to the emergence of a composition with more disorganized structures, which is attributed to the increase in kinetic energy as the cold-welding rate goes up.
4. There is no link between the stretching velocity and breaking points, which could be additional evidence for better mechanical performance of the welding joint.

With the simulation results and in consideration of the potential applications of the bearing and sleeve in nano-mechanics, there is value in this study for the mechanical working of future mechanical processing and structural nano-assembly.

## Figures and Tables

**Table 1:** Summary of the two schemes of copper bearing-sleeve at the nanoscale.

Scheme No.		Nanobearings		Welding Velocity (m/s)	Stretching Velocity (m/s)	Temperature (K)
		Inner Ring	Outer Ring			
i	I	2.0	6.0	10	30	300
	II	2.5				
	III	3.0				
	IV	3.5				
ii		2.5				150
						300
						450
						600
						750
iii				5		300
				10		
				20		
				30		
iv				10	10	
					20	
					30	

**Table 2:** Summary of the structure types of bearing-sleeve at various rates.

Stage	Velocity	Name of Types	Count of Atoms	Fraction of Atoms (%)
After equilibrium	Initial state	Unknown	11,642	20.7
		FCC	44,730	79.3
		HCP	0	0
After contact	*V*_1_ = 10 m/s	Unknown	11,390	20.2
		FCC	42,782	75.9
		HCP	2200	3.9
	*V*_2_ = 20 m/s	Unknown	12,314	21.8
		FCC	41,635	73.9
		HCP	2422	4.3
	*V*_3_ = 30 m/s	Unknown	12,430	22.0
		FCC	40,602	72.0
		HCP	3366	6.0
